# Intratumoral and peritumoral lymphatic vessel density both correlate with lymph node metastasis in breast cancer

**DOI:** 10.1038/srep40364

**Published:** 2017-01-09

**Authors:** Song Zhang, Shanhong Yi, Dong Zhang, Mingfu Gong, Yuanqing Cai, Liguang Zou

**Affiliations:** 1 Department of Radiology, Xinqiao Hospital, Third Military Medical University, Chongqing 400037, China; 2 Department of Urology, Xinqiao Hospital, Third Military Medical University, Chongqing 400037, China

## Abstract

The status of lymph node involvement is an important prognostic factor for breast cancer. However, the presence of intratumoral lymphatic vessels in primary tumor lesions and the relationship between lymphatic vessel density (LVD) and lymph node metastasis (LNM) have not been firmly established. Therefore, we performed a meta-analysis study to investigate these issues. According to the pre-established inclusion and exclusion criteria, 13 studies, involving 1029 breast cancer patients, were included in this study. Using immunohistochemical staining, intratumoral lymphatic vessels were detected in 40.07% of breast cancer patients (240/599), and peritumoral lymphatics were detected in 77.09% (397/515). All studies demonstrated that peritumoral LVD was higher than intratumoral LVD, with a pooled standard mean difference and 95% confidence interval (95% CI) of 1.75 (1.28 to 2.21). Both intratumoral LVD and peritumoral LVD positively correlated with LNM, with correlation coefficients of 0.14 (95% CI 0.05 to 0.23) and 0.31 (95% CI 0.13 to 0.49), respectively. In summary, our study reports the overall detection rate of intratumoral lymphatics and demonstrates the associations between intratumoral LVD, peritumoral LVD, and LNM in breast cancer. Additionally, controlled studies with a larger number of subjects are needed to establish these relationships.

Breast cancer is the most common malignant tumor in females. Although great efforts have been made in the field of early diagnosis and adjuvant therapy, the incidence and overall mortality of breast cancer continues to increase^1^. Since breast tumor cells commonly infiltrate into the lymphatic system, lymph node status is routinely used to identify a patient’s prognosis, tumor stage, and treatment modality^2^,^3^. Inhibition of lymph node metastasis (LNM) is a promising way to prevent distant metastasis, which has been proved by many studies^4^,^5^. However, the relationship between lymphangiogenesis and LNM remains ambiguous.

Due to the lack of specific markers, the detection of lymphatic vessels has been hampered in previous studies. Intratumoral lymphatic vessels were considered to be rare and nonfunctional due to mechanical compression^6^. With the identification of specific markers, such as podoplanin/D2–40, vascular endothelial growth factor receptor-3 (VEGFR-3), lymphatic vessel endothelial hyaluronan receptor -1 (LYVE-1) and Prox-1, many experimental and clinicopathological studies have demonstrated the existence of intratumoral lymphatics. The intratumoral lymphatics are considered to be undergoing dynamic changes that can facilitate tumor metastasis^7^. The entry of tumor cells into lymphatic vessels is promoted by lymphangiogenesis and lymphatic enlargement^8^,^9^. Therefore, lymphatic vessel density (LVD), a representation of lymphangiogenesis, can serve as an indicator of early lymphogenous spread.

Some studies have suggested that LVD is associated with an increased risk of LNM^10^,^11^; however, this conclusion is not supported by all of the published studies^12^,^13^. The evidence is limited because the published studies are observational studies and included relatively small sample sizes, which could have led to confounding factors and selection bias. Moreover, the different LVD counting methods and the varied dilutions of antibodies could have affected the conclusions. With the accumulating evidence, we conducted a meta-analysis study to investigate the overall detection rate of intratumoral lymphatics and to estimate the relationships between intratumoral LVD, peritumoral LVD and LNM in breast cancer.

## Results

### Study selection process

**Figure 1:**
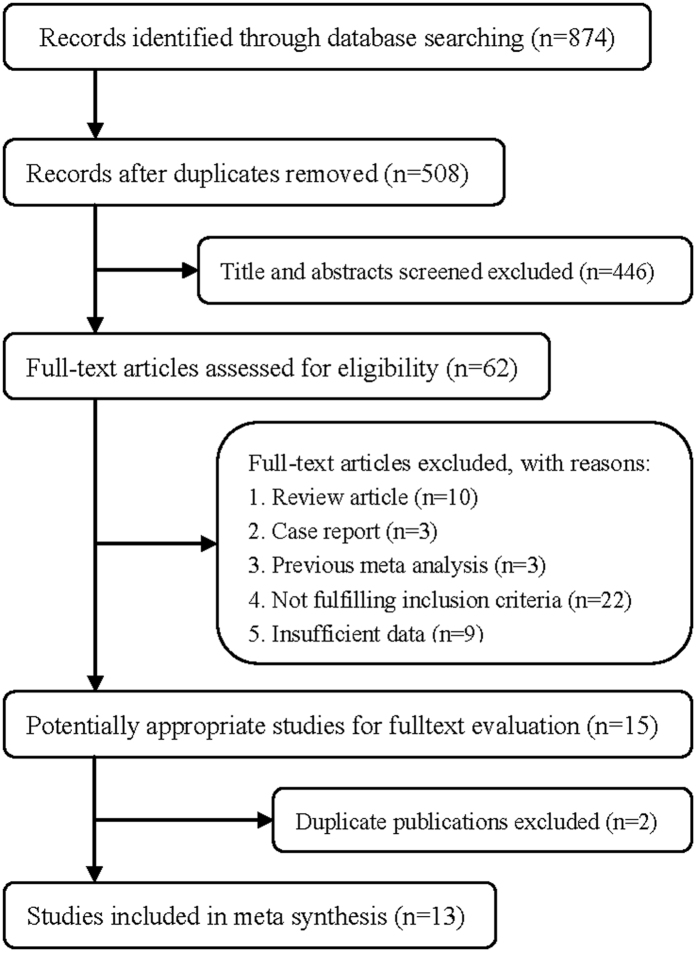
Process of study selection for the meta-analysis.

The flow chart of the article selection process is presented in Fig. 1. PubMed and Embase databases were searched to identify the relevant studies. We initially obtained 874 potential papers from the two databases, including 420 from PubMed and 454 from Embase. After screening the titles and abstracts, most of them were excluded, either because of duplicate publications, if they were letters or reviews, or did not distinguish between intratumoral LVD and peritumoral LVD. Finally, 13 papers were adopted according to the inclusion criteria.

### Characteristics of the included studies

The details of the included studies are exhibited in Table 1^10^,^11^,^12^,^13^,^14^,^15^,^16^,^17^,^18^,^19^,^20^,^21^,^22^. The publication years ranged from 2005 to 2014. A total of 1,029 breast cancer patients, ranging from 26 to 89 years old (except two studies that did not indicate the age^19^,^20^), were included in this study. All patients underwent surgical treatments and immunohistochemical examinations with D2–40/podoplanin antibodies. Intratumoral LVD and peritumoral LVD were determined by counting the number of lymphatic vessels using the high magnification field under a microscope. All studies reported sufficient sample sizes, ranging from 25 to 177 patients.

### Data analysis

**Figure 2:**
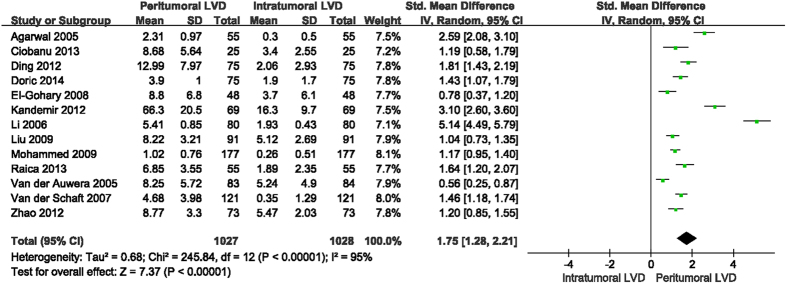
Forest plot of the standard mean differences between peritumoral LVD and intratumoral LVD in breast cancer.

Among the 13 studies included, seven^10^,^11^,^13^,^14^,^16^,^17^,^21^ reported the detection rate of intratumoral lymphatics, with an overall rate of 40.07% (240/599). Six of the included studies^10^,^11^,^13^,^14^,^16^,^17^ reported the detection rate of peritumoral lymphatics, with an overall rate of 77.09% (397/515). All 13 studies were used to assess the differences between intratumoral LVD and peritumoral LVD. The values of intratumoral and peritumoral LVD and the pooled SMD value with 95% CI are presented in Fig. 2. Despite significant heterogeneity (*P* < 0.05, I^2^ = 95%), all studies indicated that peritumoral LVD values were higher than intratumoral LVD, with a pooled SMD of 1.75 (95% CI 1.28 to 2.21). The random-effects model was used to combine the SMD values because of significant heterogeneity.

**Figure 3:**
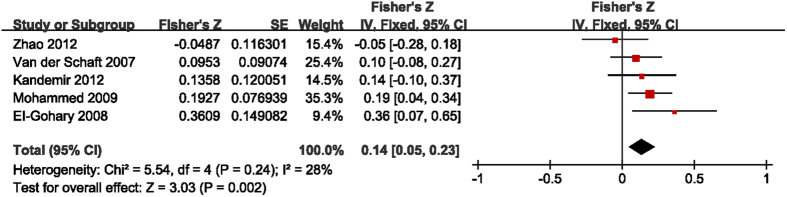
Forest plot of the Fisher’s Z values for the correlation between intratumoral LVD and LNM in breast cancer.

**Figure 4:**
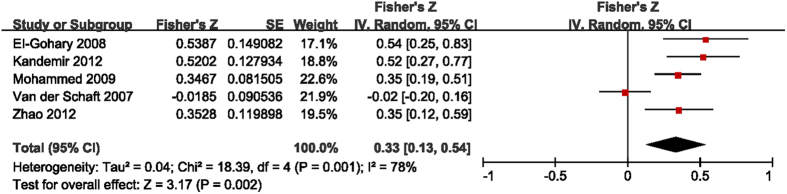
Forest plot of the Fisher’s Z values for the correlation between peritumoral LVD and LNM in breast cancer.

The studies that include detailed data of intratumoral and peritumoral LVD and the presence of LNM were selected to investigate the interrelationships between them^10^,^11^,^12^,^13^,^17^. The results presented with means and standard deviations, or two by two frequency tables, were transformed to obtain the *r* values. The Fisher’s Z transformation was used to convert *r* values to Z values. The main outcomes are summarized in Figs 3 and 4. The pooled Fisher’s Z values for the relationships between intratumoral LVD and LNM and between peritumoral LVD and LNM were 0.14 (95% CI 0.05 to 0.23, I^2^ = 28%, P = 0.002, Fig. 3) and 0.33 (95% CI 0.13 to 0.54, I^2^ = 78%, P = 0.002, Fig. 4), respectively. Finally, the pooled Fisher’s Z values were converted back to *r* values by inverse Fisher’s Z transformation. Both intratumoral LVD (r = 0.14, 95% CI 0.05 to 0.23) and peritumoral LVD (r = 0.31, 95% CI 0.13 to 0.49) were positively correlated with LNM in breast tumors.

### Sensitivity analysis and publication bias

**Figure 5:**
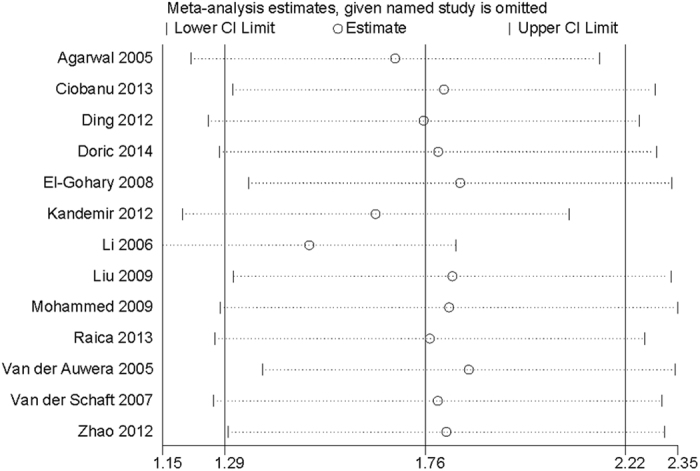
Plot of the included studies for sensitivity analysis.

**Figure 6:**
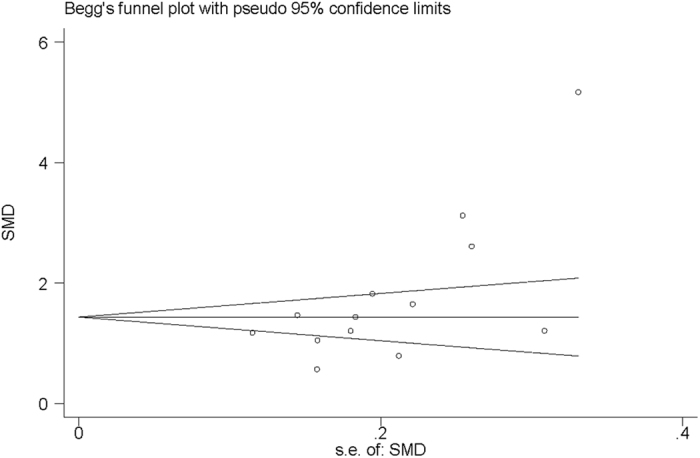
Begg’s funnel plot of the included studies for publication bias.

To evaluate the stability of the results, a sensitivity analysis was performed using the random-effects model. Sensitivity analysis, by repeatedly analyzing the data after removing individual studies in turn, demonstrated that no studies were responsible for the disproportionate influence on the pooled estimate (Fig. 5). Begg’s funnel plot of the SMD against the standard error of SMD showed substantial asymmetry (Fig. 6). Egger’s regression test showed evidence of publication bias (*P* = 0.017).

## Discussion

The current meta-analysis included 13 observational studies with an overall population of 1029 breast cancer patients. By immunohistochemical staining with D2–40/podoplanin antibodies, 40.07% of the specimens exhibited intratumoral lymphatics, and 77.09% of the specimens showed peritumoral lymphatics. Peritumoral LVD is significantly higher than intratumoral LVD in breast cancer (*P* < 0.05). The positive correlation between peritumoral LVD and LNM was moderately stronger than that of intratumoral LVD and LNM. However, there was substantial evidence of heterogeneity among these studies. By conducting a sensitivity analysis, two studies were identified to be the main source of heterogeneity^17^,^20^.

Although the existence of peritumoral lymphatics is well recognized, the presence of intratumoral lymphatics is a hotly debated issue in solid tumors, particularly in breast cancer^6^,^23^,^24^. Initial studies reported that breast cancers did not have intratumoral lymphatics^25^, owing to the increased interstitial pressure created by the proliferating cancer cells^26^. Williams *et al*.^6^ and Vleugel *et al*.^24^ failed to detect lymphangiogenesis in breast cancer by using LYVE-1 as a marker of lymphatic vessels. Using the new specific markers of lymphatic vessels, such as D2–40 and podoplanin, recent studies demonstrated that intratumoral lymphatics are detectable^11^,^27^. Intratumoral lymphatics were generally detected in 40.07% of breast cancer specimens, and the detection rate of peritumoral lymphatics was 77.09%. Moreover, intratumoral lymphatics are believed to be functional, as tumor cells have been observed to flow within the vessels^28^.

In addition, our study shows that peritumoral LVD is significantly higher than intratumoral LVD, with a pooled SMD of 1.75 (95% CI 1.28 to 2.21). The result is supported by all included studies. Peritumoral LVD is also reported to be higher than that of normal and benign breast lesions^10^,^12^,^22^. However, the comparison between intratumoral LVD and normal LVD cannot draw a consistent conclusion. Agarwal *et al*.^22^ and Van der Auwera *et al*.^21^ claimed that intratumoral LVD of breast cancer was lower than that of normal or benign breast lesions. In contrast, other studies indicated there were no differences between them^10^,^12^. These contradicting results might be due to the different locations of tumor lymphatic vessels used to define the term of “intratumoral lymphatic vessels”. Van der Auwera *et al*.^21^ regarded intratumoral lymphatics as any vessels within the tumor area, either in the inner core or periphery. Another study considered intratumoral lymphatics as vessels present only among tumor cells^13^. However, in Mohammed *et al*.’s study^11^, intratumoral lymphatic vessels referred to the vessels within the inner 2/3 core of the tumor lesion. Due to the lack of studies, the comparisons between intratumoral LVD, peritumoral LVD and the LVD of normal or benign breast lesions were not conducted in this meta-analysis study. In addition, the inconsistent definitions of “intratumoral lymphatic vessels” could also impact the comparison between intratumoral LVD and peritumoral LVD. A larger number of strict standardized studies is needed.

It is well known that blood vessel density, an indicator of tumor angiogenesis, is closely associated with the clinicopathological outcomes of breast cancer^29^. The methods used for assessing angiogenesis are usually used to measure the lymphangiogenesis of breast cancer as well^21^,^30^. Many studies have demonstrated the associations between peritumoral LVD and tumor grade, tumor stage, lymphatic invasion, LNM, and overall survival in breast cancer^10^,^31^,^32^. However, the relationship between intratumoral LVD and clinicopathological behavior is still uncertain. Our study not only demonstrates a positive association between intratumoral LVD and LNM in breast cancer but also reveals that peritumoral LVD has a moderately stronger correlation with LNM than that of intratumoral LVD. These results suggest that peritumoral lymphatic vessels have a more important effect on metastatic dissemination in breast cancer.

Although the current meta-analysis study has some definite strengths, some limitations should be considered. All included studies were observational studies with relatively small sample sizes, and several studies^6^,^23^ were excluded due to lack of data on intratumoral LVD or peritumoral LVD. Thus, recall bias and selection bias are inevitable. In addition, the unmeasured or inadequately measured factors, such as patient sources, histological types, antibody categories and antibody dilutions, could confound the results. Moreover, different counting methods for lymphatic vessels, such as the number of different hotspots (ten^17^, three^21^, and five^31^), magnification (100×^11^, 200×^31^, 400×^17^), and measuring unit (vessels/mm^2^ 11, vessels/area^21^), were used in different studies. The values of intratumoral LVD and peritumoral LVD varied notably, resulting in the significant heterogeneity. Therefore, studies with a larger sample size and more standardized methods are required to assess intratumoral LVD and peritumoral LVD.

In conclusion, the study demonstrates the existence of intratumoral lymphatic vessels. Although the overall detection rate of intratumoral lymphatic vessels is lower than that of peritumoral lymphatic vessels, it does not change the fact that they are present and constitute a risk factor for tumor metastasis. Both intratumoral LVD and peritumoral LVD are correlated with the increasing risk of LNM, and peritumoral LVD exhibits a moderately stronger correlation with the increasing risk of LNM than that of intratumoral LVD. It might provide a potential target to prevent lymphangiogenesis and lymphatic metastasis in breast cancer.

## Methods

### Search strategy

Two independent observers searched the databases of PubMed and Embase. The databases were searched using the following Medical Subject Heading (MeSH) terms or keywords: “breast cancer OR breast carcinoma OR breast neoplasms” AND “lymphatic vessel density OR lymphatic microvessel density OR LVD OR LMVD OR lymphangiogenesis” with no restrictions. All abstracts that indicated LVD assessment in breast cancer, no matter prospective or retrospective, were chosen for further consideration. The reference lists of all selected papers and abstracts were also screened. If it was necessary, we contacted the authors of the original studies for the required data. The search was ended on April 8, 2016.

### Inclusion and exclusion criteria

All studies that met the following criteria were included: (1) patients with breast cancer at any age; (2) a sample size larger than 10 patients; (3) no neoadjuvant chemotherapy or radiotherapy administered before the surgical treatment; and (4) specimens stained with the immunohistochemical method. Studies were excluded if they included the following: (1) review articles, case reports, meeting abstracts, or animal studies; (2) an examination of the total LVD of breast tumor lesions without distinguishing intratumoral LVD and peritumoral LVD; or (3) patients previously diagnosed with other diseases that could lead to LNM. Two independent authors followed the above inclusion and exclusion criteria to review the studies. When two or more articles reported duplicated data, we included the study with the most-recent data, the largest dataset, or the most relevant data. In cases of disputes, a third reviewer assessed the study to obtain a consensus.

### Data extraction and quality assessment

Two authors independently checked each item mentioned by the publications and discussed the data that was extracted. The information extracted from each study was summarized in a table and included the following items: first author’s name, publication year, country, number of patients (size), age, type of breast cancer involved, antibody and dilution, intratumoral LVD and detection rate, peritumoral LVD and detection rate. Two authors conducted the quality assessment based on the criteria of the Newcastle-Ottawa Quality Assessment scale (NOS)^33^, which evaluates the methodology in observational studies.

### Statistical analysis

Spearman correlation coefficients (*r*) were used for the meta-analysis because some variables in the original studies were log-transformed before analysis^34^. The Fisher’s Z transformation was used to convert *r* values to Z values into a normal distribution^34^. The standard mean differences (SMDs) and Z values with 95% CIs were combined by RevMan5.3 software. Homogeneity tests were performed with the Q statistic and I^2^ statistic. A random-effects model or, in the absence of heterogeneity, a fixed-effects model was used to combine the SMDs and Z values with 95% CIs. The pooled *r* values and 95% CIs were obtained from the inverse Fisher’s Z transformation. Additionally, we conducted a sensitivity analysis by STATA 12.0 software to investigate the influence of a single study on the overall result by omitting each study in turn. Publication bias was detected by Begg’s and Egger’s test. In this study, *P* < 0.05 was considered statistically significant.

## Additional Information

**How to cite this article**: Zhang, S. *et al*. Intratumoral and peritumoral lymphatic vessel density both correlate with lymph node metastasis in breast cancer. *Sci. Rep.* **7**, 40364; doi: 10.1038/srep40364 (2017).

**Publisher's note:** Springer Nature remains neutral with regard to jurisdictional claims in published maps and institutional affiliations.

## Figures and Tables

**Table 1:** Main characteristics and results of the included studies.

Author, Year, Country	Size	Age	Tumor type	Antibody and dilution	Intratumoral LVD and detection rate	Peritumoral LVD and detection rate
Doric, 2014, Bosnia and Herzegovina^14^	75	59 (37–87)^a^	invasive BC	D2–40 (1:100)	1.9 ± 1.7 (27/75)	3.9 ± 1.0 (75/75)
Raica, 2013, Italy^15^	55	26–81^b^	ductal invasive BC	D2–40 (NG)	1.89 ± 2.35 (NG)	6.85 ± 3.55 (NG)
Ciobanu, 2013, Romania^16^	25	58 (45–69)^c^	lobular invasive BC	D2–40 (1:100)	3.40 ± 2.55 (18/25)	8.68 ± 5.64 (19/25)
Zhao, 2012, China^12^	73	53.8 (29–75)^a^	ductal invasive BC	D2–40 (1:25)	5.47 ± 2.03 (NG)	8.77 ± 3.30 (NG)
Kandemir, 2012, Turkey^17^	69	54.8 (39–85)^a^	ductal invasive BC	D2–40 (1:50)	16.3 ± 9.7 (18/69)	66.3 ± 20.5 (25/69)
Ding, 2012, China^18^	75	52.1 (42–63)^a^	ductal invasive BC and Paget disease	D2–40 (NG)	2.06 ± 2.93 (NG)	12.99 ± 7.97 (NG)
Mohammed, 2009, UK^11^	177	57 (32–70)^c^	invasive BC	D2–40 (1:100)	0.26 ± 0.51 (73/177)	1.02 ± 0.76 (177/177)
Liu, 2009, China^19^	91	NG	invasive BC	D2–40 (1:100)	5.12 ± 2.69 (NG)	8.22 ± 3.21 (NG)
EI-Gohary, 2008, USA^10^	48	64 (27–89)^a^	invasive BC	D2–40 (1:50)	3.7 ± 6.1 (24/48)	8.8 ± 6.8 (46/48)
Van der Schaft, 2007, Netherlands^13^	121	61.4 ± 12.2^d^	ductal invasive BC	Podoplanin (NG)	0.35 ± 1.29 (12/121)	4.68 ± 3.98 (55/121)
Li, 2006, Japan^20^	80	NG	ductal invasive BC	D2–40 (1:100)	1.93 ± 0.43 (NG)	5.41 ± 0.85 (NG)
Agarwal, 2005, USA^22^	55	53 (35–72)^a^	invasive BC	D2–40 (1:40)	0.3 ± 0.5 (NG)	2.31 ± 0.97 (NG)
Van der Auwera, 2005, Belgium^21^	85	25.6–83.2^b^	inflammatory and non-inflammatory BC	D2–40 (1:20)	5,24 ± 4.90 (68/84)	8.25 ± 5.72 (NG)

**Note:** a: mean (range); b: range; c: median (range); d: mean ± SD; BC: breast cancer; LVD: lymphatic vessel density; NG: not given.
